# An exploratory randomized-controlled trial of the efficacy of the Src-kinase inhibitor saracatinib as a novel analgesic for cancer-induced bone pain

**DOI:** 10.1016/j.jbo.2019.100261

**Published:** 2019-09-18

**Authors:** Sarah Danson, Matthew R Mulvey, Lesley Turner, Janet Horsman, KJane Escott, Robert E Coleman, Sam H Ahmedzai, Michael I Bennett, David Andrew

**Affiliations:** a Academic Unit of Clinical Oncology, Sheffield Experimental Cancer Medicine Centre, Weston Park Hospital, University of Sheffield, UK; b Academic Unit of Palliative Care, Leeds Institute of Health Sciences, University of Leeds, UK; c Emerging Innovations Unit, BioPharmaceuticals R & D, AstraZeneca, Cambridge, UK; d Palliative Medicine, University of Sheffield, UK; e School of Clinical Dentistry, University of Sheffield, UK

**Keywords:** Cancer pain, Src inhibitor, Metastasis, Clinical trial, Saracatinib

## Abstract

Pain is a major symptom of bone metastases from advanced cancer and represents a clinical challenge to treat effectively. Basic neurobiology in preclinical animal models implicates enhanced sensory processing in the central nervous system, acting through *N*-methyl-D-aspartate (NMDA) glutamate receptors, as an important mechanism underpinning persistent pain. The non-receptor tyrosine kinase Src is thought to act as a hub for regulating NMDA receptor activity and the orally available Src inhibitor saracatinib has shown promise as a potential analgesic in recent animal studies. Here we tested the efficacy of saracatinib as a novel analgesic in an exploratory phase II randomized controlled trial on cancer patients with painful bone metastases. Twelve patients completed the study, with 6 receiving saracatinib 125 mg/day for 28 days and 6 receiving placebo. Pharmacokinetic measurements confirmed appropriate plasma levels of drug in the saracatinib-treated group and Src inhibition was achieved clinically by a significant reduction in the bone resorption biomarker serum cross-linked C-terminal telopeptide of type I collagen. Differences between the saracatinib and placebo groups self-reported pain scores, measured using the short form of the Brief Pain Inventory, were not clinically significant after 4 weeks of treatment. There was also no change in consumption of maintenance analgesia in the saracatinib-treated group and no improvement in Quality-of-Life scores. The data were insufficient to demonstrate saracatinib has efficacy as analgesic, although it may have a role as an anti-bone resorptive agent.

## 1 Introduction

Pain from bone metastases is a common feature of advanced cancer, occurring in approximately 70% of patients with late-stage disease [1,2]. The four commonest cancers worldwide (lung, breast, bowel, prostate [3]) all have a predilection to spread to bone, resulting in increased morbidity, not only due to pain, but also from fractures, spinal cord compression and hypercalcaemia (skeletal-related events). The innervation of bone and possible neurobiological mechanisms of bone pain have been comprehensively covered in a recent review [4], but an emerging feature of cancer-induced bone pain is that it has features of both neuropathic pain, due to damage to peripheral nerve fibres from the growing tumour, and inflammatory pain, due to tissue acidosis and local chemokines/cytokines. Chronic pain, including bone pain, is also associated with changes in central neural processing of nociceptive information that results in amplified behavioural responses (central sensitization [5]). Activation of C-fibres from deep tissues *e.g.* joint or muscle is thought to be a much more effective driver of central sensitization than stimulation of cutaneous afferents [6].

The consensus of preclinical laboratory studies is that synaptic plasticity in spinal cord neurons, particularly increased glutamatergic excitatory neurotransmission, is thought to be a major mechanism underlying central sensitization that contributes to pain hypersensitivity [5,[7], [8], [9]]. This form of synaptic plasticity involves activation of G-protein coupled membrane receptors and intracellular signaling pathways that are thought to converge on the protein tyrosine kinase Src [10,11]; activation of spinal *N*-methyl-D-aspartate (NMDA) glutamate receptors follows, with phosphorylation of the GLUN1 and GluN2B subunits being the key molecular drivers [5,[10], [11], [12], [13], [14]]. Blocking Src anchoring with the NMDA receptor resulted in reduced inflammatory and neuropathic pain hypersensitivity and Src^−/−^ knockout mice showed diminished central sensitization-mediated nociceptive behaviours but retained normal nociceptive processing [15]. Controlling Src activity might therefore be key to reducing NMDA receptor activation and could represent a novel analgesic mechanism.

Our recent preclinical studies have shown that the orally-active Src inhibitor saracatinib (from AstraZeneca, UK) has potential use as an analgesic in cancer-induced bone pain, as it reversed some of the pain behaviours evoked by intra-tibial injection of mammary cancer cells in rats [16]. Saracatinib also inhibits osteoclastic bone resorption [16], [17], [18], which could be useful in the management of patients with skeletal metastases. In the current study we have tested whether saracatinib has analgesic efficacy in cancer patients with bone pain due to metastatic disease.

## 2 Methods

Prior to opening for recruitment, the study was registered on the European Clinical Trials database (EudraCT number 2013-00250562). A favourable ethical opinion was obtained from the local office (Yorkshire & The Humber) of the National Research Ethics Committee of the UK Health Research Authority (13/YH/0623) and clinical trial authorization was obtained from the UK Medicines and Healthcare Regulatory Authority (CTA number: 21304/0249/001-001).

### 2.1 Participants and recruitment

Participants were recruited from either out-patient clinics in a specialized Oncology Hospital (Weston Park Hospital, Sheffield UK) or from a palliative care Hospice (St. Gemma's Hospice, Leeds UK). Potential recruits were identified by screening clinical records to identify patients with bone metastases (primary screen) and then at individual consultations with their Oncologist/Palliative Care physician (secondary screen) to identify patients that had sub-optimal control of bone pain with their existing medication. There were no limits on the type or dose of existing pain relief medications that patients were taking to be eligible for recruitment and patients taking maintenance chemotherapy were also able to participate. Patients that had previous/planned radiotherapy to the site of their pain had to be at least one month post-treatment to be eligible for recruitment and no new treatments/medications were permitted during the study.

Inclusion criteria were: (1) cytologically or histologically confirmed solid tumours or multiple myeloma with painful bone metastases and poor control of bone pain in spite of pain medication. Prostate cancer patients without histology or cytology were eligible for recruitment if their prostate specific antigen was >100 ng/mL at diagnosis and a bone scan confirmed skeletal metastases; (2) baseline average pain ≥2 and ≤9 on a 0–10 numerical scale recorded on at least two separate days using the Brief Pain Inventory-Short Form; (3) WHO performance status ≤2; (4) age ≥16 years; (5) ability to take and absorb oral medications; (6) able to give written informed consent and willing to follow the study protocol; (7) adequate baseline haematological, hepatic and renal function, which was defined as: Haemoglobin >9.0 g/dL (including after transfusion), absolute neutrophil count ≥ 1.5 × 10^9^/L, platelet count ≥100 × 10^9^/L, bilirubin ≤1.5 x upper limit of normal (ULN), alanine transaminase or aspartate transaminase ≤2.5 x ULN (≤ 5 x ULN if liver metastases), creatinine ≤1.5 x ULN.

Exclusion criteria were: (1) life expectancy <3 months; (2) previous or planned radiotherapy at site of pain within one month of first dose; (3) unstable cardiac disease in last 3 months; (4) history of interstitial lung disease; (5) unable to discontinue any medication with known moderate or potent inhibitory effect on CYP3A4, or is a substrate of CYP3A4; (6) concomitant cytotoxic chemotherapy unless established on maintenance treatment for > 6 weeks (not in a clinical trial); (7) unable to understand written or spoken English.

### 2.2 Study design, objectives and sample size

The study was a two-centre, parallel group randomised controlled phase II trial designed to assess whether the Src kinase inhibitor saracatinib had efficacy as an analgesic when compared to placebo using cancer patients’ self-reported pain scores. The primary objective was to determine if patients’ self-reported pain scores were significantly lower (pain score reduced by 2 or more on a 0–10 point scale) after 4 weeks of treatment with saracatinib than with placebo, using questions 3 (worst pain in last 24 h), 4 (least pain in last 24 h), 5 (average pain), and 6 (pain right now) of the Brief Pain Inventory - Short Form (BPI-SF [19]). Secondary objectives were (i) to determine if concomitant analgesic drug usage decreased with saracatinib; (ii) to determine if pain-related symptoms and quality of life were improved by saracatinib using the BPI-SF and the European Organisation for Research and Treatment of Cancer QLQ-C30 (v 3.0) [20] and BM-22 [21] questionnaires; (iii) to determine whether bone turnover was reduced by saracatinib in patients already taking bone anti-resorptive medications such as bisphosphonates or RANK ligand-inhibitors.

To determine if analgesic drug usage decreased when patients took saracatinib, analgesic usage data for the previous 24 h period was collected at screening and each clinic visit. The doses of all opioids were converted to an overall equivalent dose of oral morphine (oral morphine equivalent, OME). Oral morphine equivalent at the start of the study was compared to OME at the end to determine whether this changed whilst on treatment, the rationale being that if the study medication provided additional pain relief then patients might reduce their dose of other pain medications. Pain-related symptoms and quality of life at the beginning and end of the study were compared using summary data scores from the QLQ-C30 questionnaire and the painful characteristics and painful sites scores from the QLQ-BM22 questionnaire. Bone turnover was assessed by measuring markers of bone resorption (cross-linked C-terminal telopeptide of type I collagen, sCTX; and cross-linked N-terminal telopeptide of type I collagen/creatinine ratio; uNTX/Cr) and bone deposition (N-terminal propeptide of type I procollagen (PINP) in either serum (sCTX, PINP) or urine (uNTX/CR) samples [18].

For an exploratory study such as this, a group size of *n* = 12 is thought to be sufficient [22]. Using *n* = 12/group and a population standard deviation of 2.6 points in pain scores in patients with cancer pain (AstraZeneca study D8180C00034, unpublished data) with a minimally clinically important difference of 2-points [23] and a one-sided significance level of *α* = 10%, (acceptable as only a reduction in pain scores was desirable and the study was exploratory) gives a power of 72%. By comparison, a two-sided significance level of *α* = 5% gives only 50% power with the same group size.

### 2.3 Protocol

Patients that wished to participate in the study were screened to ensure that they satisfied all of the inclusion criteria and none of the exclusion criteria. Screening was done by experienced research nurses 7–14 days prior to randomization, and as well as confirming eligibility criteria, baseline samples for sCTx, PINP and uNTX/CR were taken and the BPI-SF, EORTC QLQ-C30 and BM22 questionnaires administered. To confirm that baseline pain scores were within the eligibility criteria, each potential recruit completed a daily BPI-SF questionnaire for 7 days and mean pain scores calculated using question 5 (pain on average). Patients that were eligible to enter the trial and who wished to participate gave informed written consent and were randomized to either saracatinib or placebo. Patients were randomized on a 1:1 basis using block randomization with a block size of 4; the randomization schedule was created by the company that prepared the treatment kits (Almac, Craigavon, Northern Ireland, UK). Each randomized patient was assigned a unique code that was replicated on individual patient treatment kits; the patient, the Investigator that randomized the patient and the pharmacist that dispensed the treatment kit were all blinded to the treatment assigned. Randomisation codes were assigned strictly sequentially and if a subject withdrew from the study before completion their randomization number could not re-used. At randomization a venous blood sample was taken as a baseline for pharmacokinetic studies.

Patients randomized to saracatinib received 125 mg once per day for 28 days in tablet form. This dose of saracatinib is lower than the maximum tolerated dose in patients with advanced solid malignancies (175 mg) and has an emerging safety profile that was considered to be acceptable for testing in this patient population with appropriate safety monitoring during the trial [24]. Patients were advised to take a single tablet at approximately the same time each day with food. Missed doses were not to be ‘made-up’ and if a patient vomited after taking a tablet the dose was not replaced. Compliance was assessed with a diary card and by counting the remaining tablets in the bottle that was returned at the end of treatment. Treatment was administered for 28 days unless: (1) the patient asked to be withdrawn, (2) pain control deteriorated during the trial requiring radiotherapy (3) the patient experienced unacceptable adverse effects. Treatment could be continued beyond 28 days of dosing at the discretion of the Investigator if the patient received benefit from treatment (average pain scores reduced by ≥2 points on a 0–10 scale) and there were no grade 3 or greater drug-related adverse events.

Treatment commenced at randomization (day 1) and patients completed the BPI-SF daily for 7 days after 1 week on study (days 8–14). At a clinic visit on day 15, performance status and haematological/hepatic/renal function were checked to confirm patients were still eligible to participate. Blood and urine samples were taken for sCTX, PINP, uNTX/Cr and pharmacokinetic testing and the BPI, EORTC QLQ-C30 and BM22 questionnaires administered. Analgesic usage was reviewed, any changes since screening documented and OME calculated for the previous 24 h. Adverse events were also documented. Patients remained on treatment for 2 more weeks and completed the BPI-SF daily during the last week of the study (days 22–28). A final clinic visit occurred on day 29; as before performance status and haematological/hepatic/renal function were checked, blood and urine samples were taken for sCTX, PINP and uNTX/Cr analysis, the BPI-SF, QLQ-C30 and BM22 questionnaires administered and analgesic usage was reviewed, any changes since screening documented and OME calculated for the previous 24 h. Adverse events were also documented. A telephone interview 30 days after the last clinic visit was performed to identify any further adverse events that occurred after stopping treatment.

#### 2.3.1 Sample analysis

Blood samples for haematological, hepatic and renal function were analysed immediately after collection for saracatinib safety monitoring; samples for bone biomarker turnover and pharmacokinetic analysis were prepared and frozen at -80 °C then analysed in batches. Serum (for sCTX and PINP measurement) was prepared from venous blood that was allowed to clot at room temperature for at least 30 min, centrifuged at 1300 g for 15 min and aliquots frozen. Plasma (for pharmacokinetic studies) was prepared by centrifuging venous blood collected in lithium heparin tubes at 1000 g for 10 min within 30 min of collection, aliquoted and frozen. Urine samples (1 mL volume, for uNTX/Cr) were frozen until analyzed.

Saracatinib levels were measured in plasma samples using liquid chromatography-mass spectrometry (Charles River, Tranent, UK) using an ABI5000 mass spectrometer (AB Sciex, Ontario, Canada). The lower limit of the assay was 0.5 ng/mL and the upper limit was 500 ng/mL. Bone biomarkers were analysed as described previously [18].

## 3 Results

**Fig. 1:**
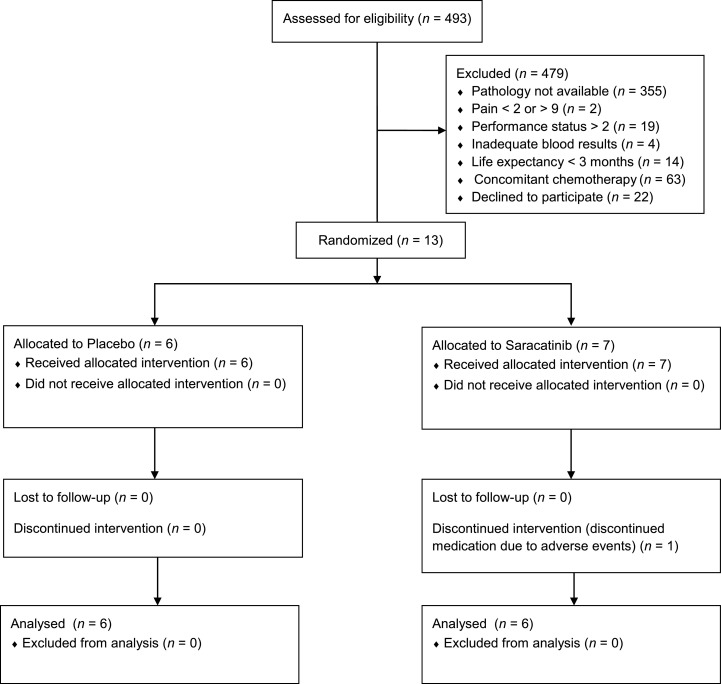
Participant flow diagram (CONSORT).

During the period September 2014–August 2017, 36 patients were identified that were eligible to participate: 15 of these gave written consent, 13 were randomized and 12 patients completed the study (Fig. 1). The commonest reasons for eligible patients to decline participation were the extra hospital visits required for the study and a 50% chance of receiving placebo. The trial ended after 3 years when the funding period ceased. Participants were allocated equally to the placebo and saracatinib groups and their demographics are shown in Table 1. The commonest histology/primary site was adenocarcinoma of the prostate (50%) and lytic lesions were the most frequent type of bone metastases identified on imaging (56%; mixed lytic-sclerotic lesions comprised 22% and sclerotic metastases were 22%). The commonest site for bone metastases was the spine (50%) followed by the pelvis (22%). Baseline average pain scores (BPI-SF question 5) from the screening process showed comparable levels of background pain in the placebo- and saracatinib-treated groups: placebo 5.0 ± 1.8 (mean ± 1 SD), saracatinib 5.2 ± 1.2, in keeping with moderate pain.

**Table 1:** Demographics of participants.

	Placebo(*n* = 6)	Saracatinib(*n* = 6)
Gender	2 female, 4 male	2 female, 4 male
Age^a^ (years)	57–71 (65 ± 5)	47–74 (61 ± 10)
Time since diagnosis^b^ (months)	4–123 (27)	2–64 (18)
Median number of bone metastases (range)	3 (1–4)	3 (1–4)
Previous radiotherapy for bone pain control	5/6	5/6
Primary tumour	Breast = 1 Lung = 2 Prostate = 3	Breast = 2 Chordoma = 1 Prostate = 3

a Age at trial entry is given as the range (mean ± 1 S.D.).

b Number of months between diagnosis of bone metastasis and starting the study is given as the range (median).

Patients randomized to saracatinib 125 mg/day had plasma concentrations of drug in the range 80–190 ng/ml (mean: 148, S.D. 38); this is comparable with other studies performed on this patient population [2,38]. There was no detectable saracatinib in plasma samples from any of the patients randomized to placebo.

### 3.1 Efficacy of saracatinib as an analgesic for metastatic bone pain

**Fig. 2:**
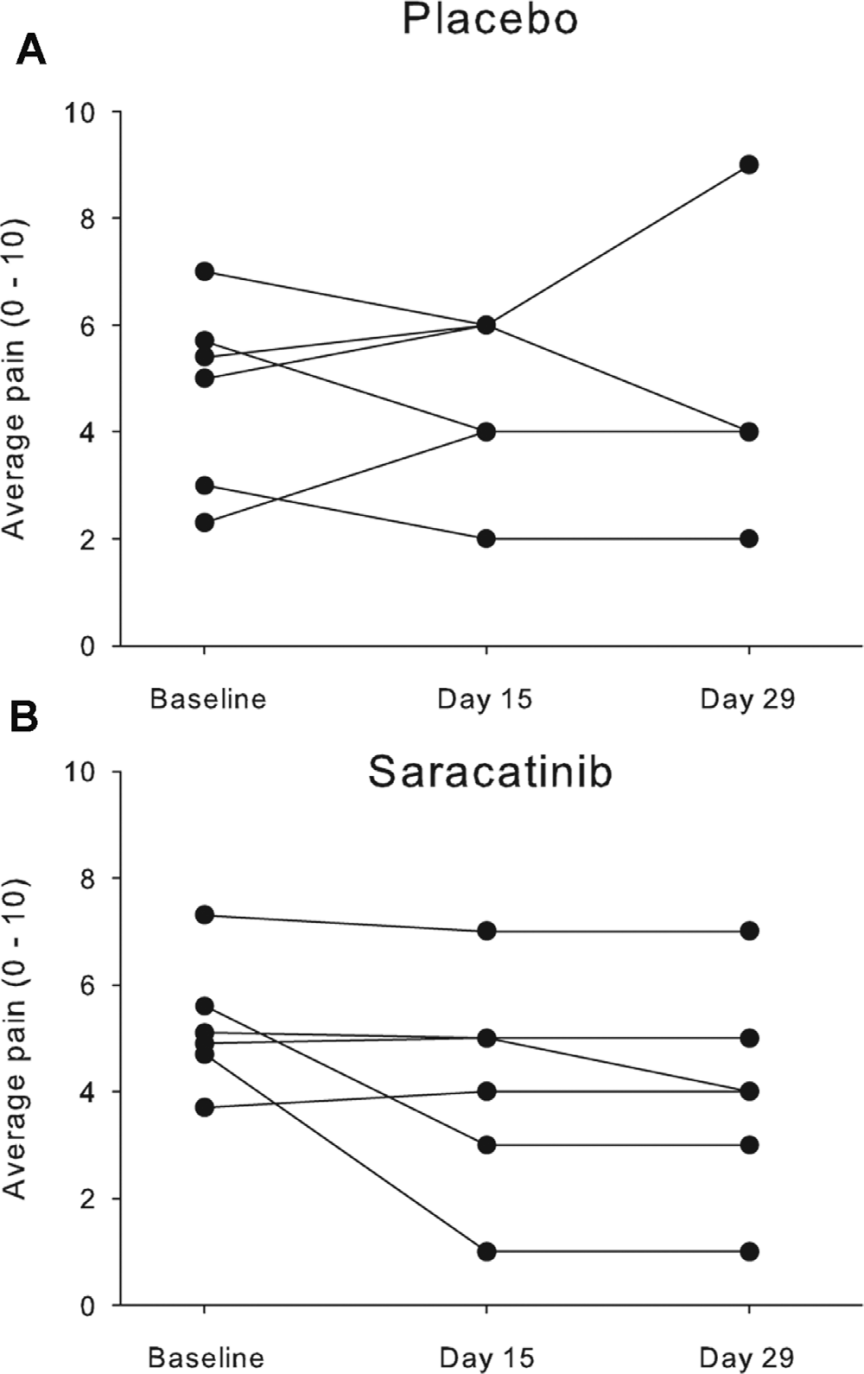
Average pain scores during the study. Patient-reported average pain scores (BPI-SF question 5, 0–10 scale) over the 4 weeks of treatment with either placebo (A) or saracatinib 125 mg/day (B).

Comparison of patients’ self-reported pain scores after 4 weeks of treatment are shown in Table 2 for worst, least, average and current pain (BPI-SF questions 3–6) and average pain scores for individual patients (BPI-SF question 5) over the 4 weeks of the study are shown in Fig. 2. As can be seen from Fig. 2, placebo-treated patients had no clear trend in pain scores over time whereas for saracatinib-treated patients, pain scores were either stable or reduced slightly over time (2/6 patients reported average pain scores reduced by ≥2, indicating a clinically -significant effect in those individuals). When self-reported pain ratings were compared (Table 2), the difference between placebo- and saracatinib-treated groups after 4 weeks of treatment was not clinically significant (< 2.0), regardless of which question was analysed. Similar results were obtained when the amount of relief obtained by pain medications was compared between the placebo- and saracatinib-treated groups (data not shown).

**Table 2:** Primary outcome.

	Placebo(*n* = 6)	Saracatinib(*n* = 6)	Difference(95% C.I.)
Worst pain in last 24 h (Q3)	6.6 ± 2.3	6.0 ± 2.4	0.6 (-2.6 to 3.9)
Least pain in last 24 h (Q4)	3.0 ± 3.1	3.2 ± 2.0	-0.2 (-2.5 to 3.2)
Average pain (Q5)	4.7 ± 2.6	4.3 ± 1.9	0.4 (-2.8 to 3.6)
Pain right now (Q6)	4.9 ± 2.6	4.5 ± 2.7	0.4 (-3.2 to 4.1)

Mean (±1 S.D.) pain scores for BPI-SF questions 3–6 after 4 weeks treatment with either saracatinib (125 mg/day) or placebo.

On the basis of improvement in pain symptoms after 4 weeks of treatment of at least 2 units on a 0–10 scale, 2 patients in the placebo-treated group and 3 patients in the saracatinib-treated group went on to extended use of study medication for up to 5 months. One of the saracatinib-treated patients initially declined extended use, but later requested extended use when her pain increased after the study ended. An unblinded pharmacist ensured that patients on extended use continued to receive the same medication that they were randomized to on day 1.

### 3.2 Effect of saracatinib on background analgesic usage

There was wide variation in OME intake across patients, which was due to the diversity of drugs that patients were prescribed for pain management as well as inter-individual differences in dosing. In the placebo-treated group mean OME at randomization was 130 ± 153 μg/day (range 3–340) and 138 ± 167 μg/day at the end of the 4 week study (range 12 – 340). Equivalent figures for the saracatinib treated group were 210 ± 352 μg/day at randomization (range 18–920) and 207 ± 327 μg/day at the completion of the study (range 5–860). The data indicate that 4 weeks of daily saracatinib treatment at a dose of 125 mg/day had no effect on patients’ consumption of regularly prescribed pain medication.

### 3.3 Effects of saracatinib on quality of life

Quality of Life was assessed using 4 scores from the QLQ-C30 and QLQ-BM22 questionnaires developed by the European Organisation for Research and Treatment of Cancer [1,4]: C30 global health-related Quality-of-Life, C30 pain items, BM22 painful sites items and the BM22 painful characteristics items. These data are shown in Table 3, and as can be seen the difference between the mean scores between the placebo- and saracatinib-treated groups were small and unlikely to be clinically -significant.

**Table 3:** Effects of saracatinib on Quality of Life measured with the QLQ-C30 and QLQ-BM22 questionnaires.

	Placebo(*n* = 6)	Saracatinib(*n* = 6)	Difference(95% C.I.)
QLQ-C30, Global health-related Quality-of-Life score, day 29	69.1 ± 9.9	66.6 ± 10.0	2.5 (-16.79 to 21.84)
QLQ-C30, Pain score, day 29	61.1 ± 9.6	58.3 ± 17.5	2.8 (-18.8 to 23.3)
QLQ-BM22, painful characteristics score, day 29	38.9 ± 14.3	44.4 ± 0	-5.6 (-28.4 to 17.3)
QLQ-BM22 painful sites score, day 29	28.3 ± 12.6	36.0 ± 18.6	-3.9 (-26.4 to 18.6)

Note that higher Global Quality-of-Life scores indicate better Quality-of-Life, while higher pain-related scores reflect worse symptoms.

### 3.4 Effects of saracatinib on biomarkers of bone turnover

**Fig. 3:**
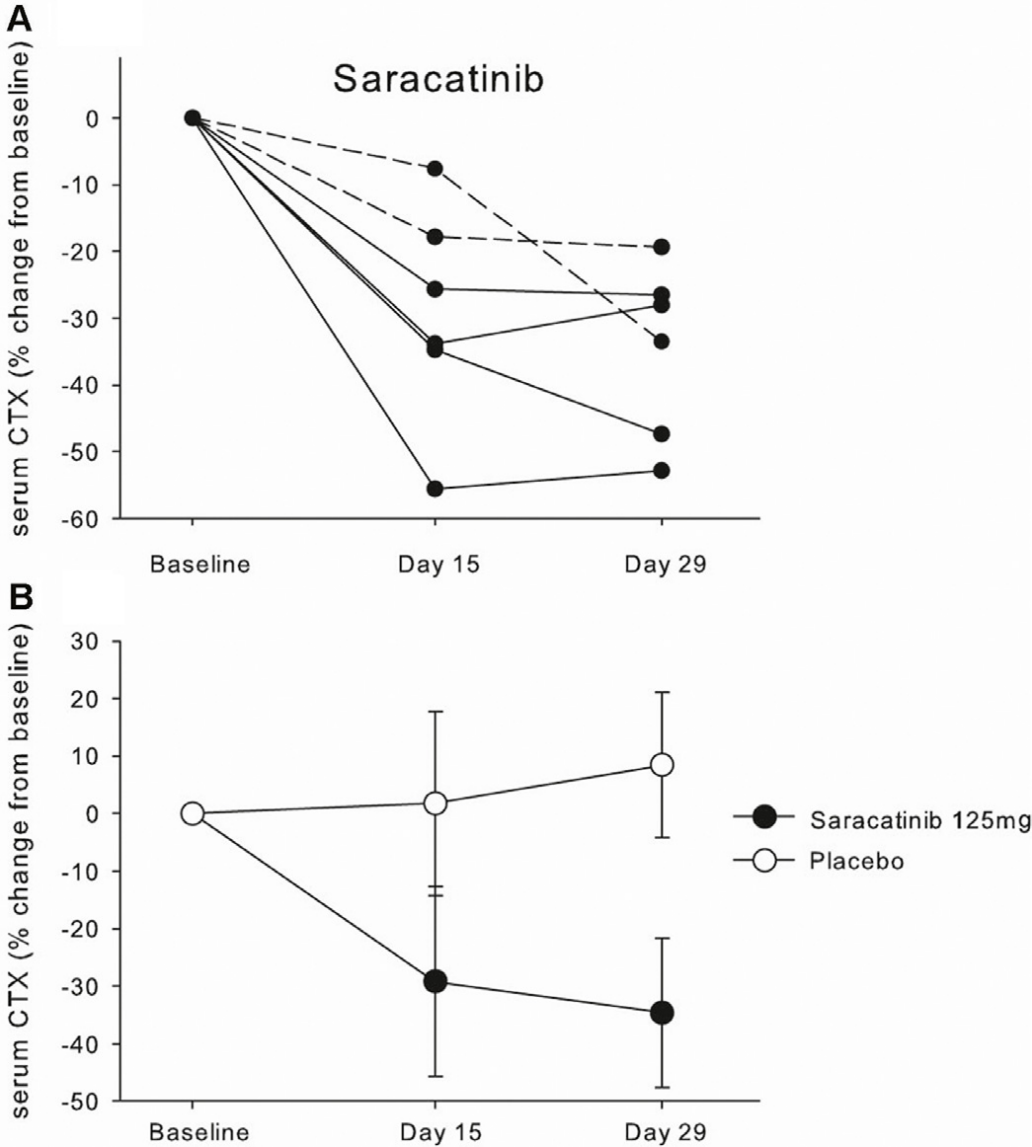
Inhibition of bone resorption by saracatinib. A. Changes in the bone resorption biomarker sCTX (serum cross-linked C-terminal telopeptide of type I collagen) from baseline in individual patients over 4 weeks of treatment; the dotted lines identify those patients that were also receiving bisphosphonate treatment. B. mean ± 1 S.D. for both saracatinib and placebo groups.

We studied two biomarkers of bone resorption, sCTX; and uNTX/Cr and one biomarker of bone deposition, PINP. Four patients in the placebo-treated group had a history of anti-resorptive medication treatment (three with bisphosphonates and one with the RANKL inhibitor denosumab) and three of these continued to receive treatment during the study (2 bisphosphate and 1 denosumab); in the saracatinib group 3 patients had a history of bisphosphonate treatment (including one who also received denosumab) and two of them continued with bisphosphonate treatment during the study. Patients taking saracatinib showed a significant reduction in levels of serum CTX over time, with a median reduction of 31% below baseline on day 29 (range 19–53%, Fig. 3; *p* < 0.01, Kruskall–Wallis ANOVA). This effect on bone resorption is in keeping with the known action on osteoclasts of Src inhibitors [17,18,25] and confirms that saracatinib inhibited Src function clinically in this patient group. Both patients in the saracatinib-treated group that were also taking concomitant bisphosphonates showed reductions in serum CTX, of 34% and 19% below baseline, indicating that saracatinib has potential to further reduce bone turnover in patients already taking anti-resorptive medication. There were no effects of saracatinib on urinary NTX/Cr ratio or on the bone formation biomarker PINP (data not shown).

### 3.5 Safety

There were 45 reported adverse events during the study, 23 in the placebo-treated group and 22 in the saracatinib-treated group. The majority of adverse events were mild (83%) and needed observation only and no intervention (CTC grade 1), 14% were grade 2 adverse events and there was one grade 3 event that required hospitalization (incoherence in a patient randomized to placebo; subsequently diagnosed as progressive brain disease and unrelated to the study medication). In the saracatinib-treated group, the majority of the adverse events (73%) affected the gastrointestinal system, with 13% being neurological and 9% fatigue, in keeping with the known adverse event profile of saracatinib [26], [27], [28], [29], [30], [31], [32], [33], [34], [35], [36], [37], [38], [39]. One patient experienced alopecia which led to her stopping the trial.

## 4 Discussion

The current study showed that 4 weeks of treatment with saracatinib did not have a clinically-significant effect on self-reported bone pain scores in patients with symptomatic skeletal metastases. The lack of analgesic efficacy of saracatinib is unlikely to be due to inadequate dosing, as plasma concentrations of drug were similar to published phase I studies [24,40] and inhibition of Src kinase was achieved on the basis of a significant reduction in sCTX levels. In addition, a dose of 125 mg saracatinib per day would be expected to give a drug concentration within cerebrospinal fluid of about 10 nM, which is considered sufficient to inhibit Src kinase within the central nervous system [40]. A limitation of this study was the small sample size per arm of the study. There were significant challenges in patient participation: only half of the required number of patients recruited, and by necessity it meant that a range of primary cancer types were studied. This limits the conclusions that can be drawn from the study, and it should be regarded as exploratory only.

Clinical studies support the contention that some of the pain symptoms that are reported by patients with bone metastases are due to changes in sensory processing within the central nervous system [41,42]. Given this, our hypothesis was that saracatinib would exert any potential analgesic activity it had by inhibiting Src activity within the central nervous system (see Introduction). In neurons, Src is part of the post-synaptic NMDA complex [10], but it is also enriched in osteoclasts [43] and platelets [44]. Our previous laboratory studies showed *in vivo* inhibition of phosphorylation of the GluN1 subunit of the NMDA receptor by saracatinib in a preclinical model of cancer-induced bone pain; saracatinib also inhibited osteoclastic bone resorption without affecting osteoblastic bone formation [16]. The results from the current study suggest that although saracatinib reduces bone resorption, either it has less impact than expected on NMDA receptor activity *in vivo*, or that central sensitisation was not the dominant pain mechanism in the patients that were recruited. It is known that osteoclastic inhibitors such as bisphosphonates have some analgesic effect [45] and inhibition of bone resorption using RANK ligand inhibitors (*e.g.* denosumab [46]) or promoting bone formation with parathyroid hormone (1–34) [47] can reduce pain from bone metastases and from osteoporotic vertebral fractures in patients. Compared to the bisphosphonate zoledronic acid, denosumab delayed the development of severe pain (scores ≥7 on a 0–10 scale) by about one month in patients with bone metastases [46]. This is presumably due to the superior bone-preserving effect of denosumab versus zoledronic acid [48], as the time to the first skeletal-related event in a study of denosumab *vs.* zoledronic acid was 6 months longer for denosumab [46]. Saracatinib appears to have the potential to inhibit tumour-related bone resorption in patients already taking anti-resorptive agents, as shown in two patients here, and in the future this might be an avenue that could be studied further.

As well as defects in pain processing [15] genetically-modified mice that have the *src* gene deleted (Src^−/−^) show abnormal development of bones and teeth with an osteopetrosis-like phenotype [49]. Multiple genes have been implicated in the development of osteopetrosis in humans and in rodents (see [50] for review), and the majority of the proteins that these genes encode for relate to the function of a proton ATPase and a chloride channel in the osteoclast ruffled membrane. The likely mechanism of saracatinib-induced inhibition of bone resorption is that Src controls the co-localization of the proton pump and the b splice variant of the chloride intracellular channel protein 5 (CLIC-5b) in osteoclasts [51] and inhibiting Src prevents channel co-localization and reduces osteoclastic bone resorption. However, the only human diseases that implicate abnormal Src function in their pathogenesis are a subset of advanced colon cancers that were metastatic to the liver [52] as well as thrombocytopenia-6 [53], and as yet there is no evidence for a role of abnormal Src function in clinical pain in patients.

## Funding

This work was funded by the UK Medical Research Council through the “Mechanisms of Human Disease” initiative (MR/K015052/1), in collaboration with AstraZeneca. AstraZeneca provided placebo and saracatinib tablets and knowledge on saracatinib. We are indebted to the patients that took part in this study.

## CRediT authorship contribution statement

**Sarah Danson:** Conceptualization, Funding acquisition, Investigation, Supervision, Writing - review & editing. **Matthew R Mulvey:** Investigation, Writing - review & editing. **Lesley Turner:** Data curation, Project administration. **Janet Horsman:** Data curation, Project administration. **KJane Escott:** Methodology, Resources, Writing - review & editing. **Robert E Coleman:** Conceptualization, Funding acquisition, Writing - review & editing. **Sam H Ahmedzai:** Conceptualization, Funding acquisition, Writing - review & editing. **Michael I Bennett:** Investigation, Project administration, Supervision, Writing - review & editing. **David Andrew:** Conceptualization, Formal analysis, Funding acquisition, Methodology, Project administration, Supervision, Writing - original draft, Writing - review & editing.

## Declaration of Competing Interest

S Danson has received fees from Amgen for giving lectures.

KJ Escott is an employee of AstraZeneca.

RE Coleman is co-inventor and patent holder of a bone biomarker under development by Inbiomotion, has received fees for advising on clinical trial protocol development from Amgen, Astellas and Boehringer Ingelheim as well as for independent medical education lectures at symposia supported by Amgen, Genomic Health and Eisai.

SH Ahmedzai has received support for undertaking cancer pain studies, giving lectures or for consultancy from Grunenthal, Menarini, Mundipharma and Pfizer

The remaining authors have no declaration of interests.

## Appendix Supplementary materials

Image, application 1

## References

[1] Coleman R.E. (2006). Clinical features of metastatic bone disease and risk of skeletal morbidity. Clin. Cancer Res..

[2] Jehn C.F., Diel I.J., Overkamp F., Kurth A., Shaefer R., Miller K., Lüftern D. (2016). Management of metastatic bone disease for diagnostics and treatment. Anticancer Res.

[3] https://www.cancerresearchuk.org/sites/default/files/cs_report_world.pdf, 2014.

[4] Mantyh P.W. (2014). The neurobiology of skeletal pain. Eur. J. Neurosci..

[5] Woolf C.J., Salter M.W. (2000). Neuronal plasticity: increasing the gain in pain. Science.

[6] Woolf C.J., Wall P.D. (1986). Relative effectiveness of C primary afferent fibers of different origins in evoking a prolonged facilitation of the flexor reflex in the rat. J. Neurosci..

[7] Schwei M.J., Honore P., Rogers S.D., Salak-Johnson J.L., Finke M.P., Ramnaraine M.L., Clohisy D.R., Mantyh P.W. (1999). Neurochemical reorganization of the spinal cord in a murine model of bone cancer pain. J. Neurosci..

[8] Urch C.E., Donovan-Rodgriguez T., Dickenson A.H. (2003). Alterations in dorsal horn neurones in a rat model of cancer-induced bone pain. Pain.

[9] Yanagisawa Y., Furue H., Kawamata T., Uta D., Yamamoto J., Furuse S., Katafuchi T., Imoto K., Iwamoto Y., Yoshimura M. (2010). Bone cancer induces a unique central sensitization through synaptic changes in a wide area of the spinal cord. Mol. Pain.

[10] Salter M.W., Kalia L.V. (2004). Src kinases: a hub for NMDA receptor regulation. Nat. Rev. Neurosci..

[11] Salter M.W., Pitcher G.M. (2012). Dysregulated Src upregulation of NMDA receptor activity: a common link in chronic pain and schizophrenia. FEBS J..

[12] Liu X.J., Salter M.W. (2010). Glutamate receptor phosphorylation and trafficking in pain plasticity in spinal cord dorsal horn. Eur. J. Neurosci..

[13] Ohnishi H., Murata Y., Okazawa H., Matozaki T. (2011). Src family kinases: modulators of neurotransmitter receptor function and behaviour. TINS.

[14] Petrenko A.B., Yamakura T., Baba H., Shimoji K. (2003). The role of *N*-methyl-D-aspartate (NMDA) receptors in pain: a review. Anesth. Analg..

[15] Liu X.J., Gingrich J.R., Vargas-Caballero M., Dong Y.N., Sengar A., Beggs S., Wang S.-.H., Ding H.K., Frankland P.W., Salter M.W. (2008). Treatment of inflammatory and neuropathic pain by uncoupling Src from the NMDA receptor complex. Nat. Med..

[16] de Felice M., Lambert D., Holen I., Escott K.J., Andrew D. (2016). Effects of Src-kinase inhibition in cancer-induced bone pain. Mol. Pain.

[17] Hannon R.A., Clack G., Rimmer M., Swaisland A., Lockton J.A., Finkelman R.D., Eastell R. (2010). Effects of the Src kinase inhibitor saracatinib (AZD0530) on bone turnover in healthy men: a randomized, double-blind, placebo-controlled multiple-ascending-dose Phase I trial. J. Bone Min. Res..

[18] Hannon R.A., Finkelman R.D., Clack G., Iacona R.B., Rimmer M., Gossiel F., Baselga J., Eastell R. (2012). Effects of Src kinase inhibition by saracatinib (AZD0530) on bone turnover in advanced malignancy in a Phase I study. Bone.

[19] Cleeland C.S., Ryan K.M. (1994). Pain assessment: global use of the brief pain inventory. Ann. Acad. Med. Singap..

[20] Aaronson N.K., Ahmedzai S., Bergman B., Bullinger M., Cull A., Duez N.J., Filiberti A., Flechtner H., Fleishman S.B., de Haes J.C.J.M., Kaasa S., Klee M., Osoba D., Razavi D., Rofe P.B., Schraub S., Sneeuw K., Sullivan M., Takeda F. (1993). The European organization for research and treatment of cancer QLQ-C30: a quality-of-life instrument for use in international clinical trials in oncology. J. Natl. Cancer Inst..

[21] Chow E., Hird A., Velikova G., Johnson C., Dewolf L., Bezjak A., Wu J., Shafiq J., Sezer O., Kardamakis D., Linden Y.v., Ma B., Castro M., Arnalot P.F., Ahmedzai S., Clemons M., Hoskin P., Yee A., Brundage M., Bottomley A., EORTC Quality of Life Group, Collaboration for Cancer Outcomes Research and Evaluation (2009). The European organization for research and treatment of cancer quality of life questionnaire for patients with bone metastases: the EORTC QLQ-BM22. Eur. J. Cancer.

[22] Julious S. (2005). Sample size of 12 per group rule of thumb for a pilot study. Pharmaceut. Statist..

[23] Farrer J.Y., Portenoy R.K., Berlin J.A., Kinman J.L., Strom B.L. (2000). Defining the clinically important difference in pain outcome measures. Pain.

[24] Baselga J., Cervantes A., Martinelli E., Chirivella I., Hoekman K., Hurwitz H.I., Jodrell D.I., Hamberg P., Casado E., Elvin P., Swaisland A., Iacona R., Taberno J. (2010). Phase I safety, pharmacokinetics and inhibition of Src activity study of saracatinib in patients with solid tumours. Clin. Cancer Res..

[25] de Vries T.J., Mullender M.G., van Duin M.A., Semeins C.M., James N., Green T.P., Everts V., Klein-Nulend J. (2009). The Src inhibitor AZD0530 reversibly inhibits the formation and activity of human osteoclasts. Mol. Cancer Res..

[26] Fury M.G., Baxi S., Shen R., Kelly K.W., Lipson B.L., Carlson D., Stambuk H., Haque S., Pfister D.G. (2011). Phase II study of saracatinib (AZD0530) for patients with recurrent or metastatic head and neck squamous cell carcinoma (HNSCC). Anticancer Res..

[27] Gangadhar T.C., Clark J.I., Karrison T., Gajewski T.F. (2013). Phase II study of the src kinase inhibitor saracatinib (AZD0530) in metastatic melanoma. Invest. New Drugs.

[28] Gubens M.A., Burns M., Perkins S.M., Pedro-Salcedo M.S., Althouse S.K., Loehrer P.J., Wakelee H.A. (2015). A Phase II study of saracatinib (AZD0530), a src inhibitor, administered orally daily to patients with advanced thymic malignancies. Lung Cancer.

[29] Gucalp A., Sparano J.A., Caravelli J., Santamauro J., Patil S., Abbruzzi A., Pellegrino C., Bromberg J., Dang C., Theodoulou M., Massague J., Norton L., Hudis C., Traina T.A. (2011). Phase II trial of saracatinib (AZD0530), an oral SRC-inhibitor for the treatment of patients with hormone receptor-negative metastatic breast cancer. Clin. Breast Cancer.

[30] Kaye S., Aamdal S., Jones R., Freyer G., Pujade-Lauraine E., de Vries E.G., Barriuso J., Sandhu S., Tan D.S., Hartog V., Kuenen B., Ruijter R., Kristensen G.B., Nyakas M., Barrett S., Burke W., Pietersma D., Stuart M., Emeribe U., Boven E. (2012). Phase I study of saracatinib (AZD0530) in combination with paclitaxel and/or carboplatin in patients with solid tumours. Br. J. Cancer.

[31] Lara P.N., Longmate J., Evans C.P., Quinn D.I., Twardowski P., Chatta G., Posadas E., Stadler W., Gandara D.R. (2009). A Phase II trial of the Src-kinase inhibitor AZD0530 in patients with advanced castration-resistant prostate cancer: a California Cancer Consortium study. Anticancer Drugs.

[32] Laurie S.A., Goss G.D., Shepherd F.A., Reaume M.N., Nicholas G., Philip L., Wang L., Schwock J., Hirsh V., Oza A., Tsao M.S., Wright J.J., Leighl N.B. (2014). A Phase II trial of saracatinib, an inhibitor of Src kinases, in previously-treated non-small cell lung cancer: the Princess Margaret Hospital Phase II consortium. Clin. Lung Cancer.

[33] Mackay H.J., Au H.J., McWhirter E., Alcindor T., Jarvi A., MacAlpine K., Wang L., Wright J.J., Oza A.M. (2012). A Phase II trial of the Src kinase inhibitor saracatinib (AZD0530) in patients with metastatic or locally advanced gastric or gastro esophageal junction (GEJ) adenocarcinoma: a trial of the PMH Phase II consortium. Invest New Drugs.

[34] McNeish I.A., Ledermann J.A., Webber L., James L., Kaye S.B., Hall M., Hall G., Clamp A., Earl H., Banerjee S., Kristeleit R., Raja F., Feeney A., Lawrence C., Dawson-Athey L., Persic M., Khan I. (2014). A randomized, placebo-controlled trial of weekly paclitaxel and saracatinib (AZD0530) in platinum-resistant ovarian, fallopian tube or primary peritoneal cancer. Ann. Oncol..

[35] Molina J.R., Foster N.R., Reungwetwattana T., Nelson G.D., Grainger A.V., Steen P.D., Stella P.J., Marks R., Wright J., Adjei A.A. (2014). A Phase II trial of the Src-kinase inhibitor saracatinib after four cycles of chemotherapy for patients with extensive stage small cell lung cancer: NCCTG trial N-0621. Lung Cancer.

[36] Posadas E.M., Ahmed R.S., Karrison T., Szmulewitz R.Z., O'Donnell P.H., Wade J.L., Shen J., Gururajan M., Sievert M., Stadler W.M. (2016). Saracatinib as a metastasis inhibitor in metastatic castration-resistant prostate cancer: a university of Chicago phase 2 consortium and DOD/PCF prostate cancer clinical trials consortium study. Prostate.

[37] Powles T., Brown J., Larkin J., Jones R., Ralph C., Hawkins R., Chowdhury S., Boleti E., Bhal A., Fife K., Webb A., Crabb S., Geldart T., Hill R., Dunlop J., Hall P.E., McLaren D., Ackerman C., Beltran L., Nathan P. (2016). A randomized, double-blind Phase II study evaluating cediranib versus cediranib and saracatinib in patients with relapsed metastatic clear-cell renal cancer (COSAK). Ann. Oncol..

[38] Reddy S.M., Kopetz S., Morris J., Parikh N., Qiao W., Overman M.J., Fogelman D., Shureiqi I., Jacobs C., Malik Z., Jimenez C.A., Wolff R.A., Abbruzzese J.L., Gallick G., Eng C. (2015). Phase II study of saracatinib (AZD0530) in patients with previously treated metastatic colorectal cancer. Invest. New Drugs.

[39] Renouf D.J., Moore M.J., Hedley D., Gill S., Jonker D., Chen E., Walde D., Goel R., Southwood B., Gauthier I., Walsh W., McIntosh L., Seymour L. (2012). A Phase I/II study of the Src inhibitor saracatinib (AZD0530) in combination with gemcitabine in advanced pancreatic cancer. Invest. New Drugs.

[40] Nygaard H.B., Wagner A.F., Bowen G.S., Good S.P., MacAvoy M.G., Strittmatter K.A., Kaufman A.C., Rosenberg B.J., Sekine-Konno T., Varma P., Chen K., Koleske A.J., Reiman E.M., Strittmatter S.M., van Dyck C.H. (2015). A phase Ib multiple ascending dose study of the safety, tolerability, and central nervous system availability of AZD0530 (saracatinib) in Alzheimer's disease. Alzheimers Res. Ther..

[41] Laird B.J.A., Walley J., Murray G.D., Clausen E., Colvin L.A., Fallon M.T. (2011). Characterization of cancer-induced bone pain: an exploratory study. Support. Cancer Care.

[42] Scott A.C., McConnell S., Laired B., Colvin L., Fallon M. (2012). Quantitative sensory testing to assess the sensory characteristics of cancer-induced bone pain after radiotherapy and potential clinical biomarkers of response. Eur. J. Pain.

[43] Horne W.C., Neff L., Chatterjee D., Lomri A., Levy J.B., Baron R. (1992). Osteoclasts express high levels of pp60c-src in association with intracellular membranes. J. Cell Bio.l.

[44] Brugge J.S., Yonemoto W., Lustig A., Golden A. (1986). Investigations of the expression of the cellular src gene product. Princess Takamatsu Symp.

[45] Wong R.K.S., Wiffen P.J. (2002). Bisphosphonates for the relief of pain secondary to bone metastases. Cochrane Database Syst. Rev..

[46] Henry D., Vadhan-Raj S., Hirsh V., von Moos R., Hungria V., Costa L., Woll P.J., Scagliotti G., Smith G., Feng A., Jun S., Dansey R., Yeh H. (2014). Delaying skeletal-related events in a randomized phase 3 study of denosumab versus zoledronic acid in patients with advanced cancer: an analysis of data from patients with solid tumors. Supp. Care Cancer.

[47] Hadji P., Zanchetta J.R., Russo L., Recknor C.P., Saag K.G., McKiernan F.E., Silverman S.L., Alam J., Burge R.T., Krege J.H., Lakshmanan M.C., Masica D.N., Mitlak B.H., Stock J.L. (2012). The effect of teriparatide compared with risedronate on reduction of back pain in postmenopausal women with osteoporotic vertebral fractures. Osteoporos Int..

[48] Lipton A., Fizazi K., Stopeck A.T., Henry D.H., Brown J.E., Yardley D.A., Richardson G.E., Siena S., Maroto P., Clemens M., Bilynskyy B., Charu V., Beuzeboc P., Rader M., Viniegra M., Saad F., Ke C., Braun A., Jun S. (2012). Superiority of denosumab to zoledronic acid for prevention of skeletal-related events: a combined analysis of 3 pivotal, randomized, phase 3 trials. Eur. J. Cancer.

[49] Soriano P., Montgomery C., Geske R., Bradley A. (1991). Targeted disruption of the c-*src* proto-oncogene leads to osteopetrosis in mice. Cell.

[50] del Fattore A., Cappariello A., Teti A. (2008). Genetics, pathogenesis and complications of osteopetrosis. Bone.

[51] Edwards J.C., Cohen C., Weibing X., Schlesinger P.H. (2006). c-Src control of chloride channel support for osteoclast HCl transport and bone resorption. J. Biol. Chem..

[52] Irby R.B., Mao W., Coppola D., Kang J., Loubeau J.M., Trudeau W., Karl R., Fujita D.J., Jove R., Yeatman T.J. (1999). Activating Src mutation in a subset of advanced human colon cancers. Nature Genet.

[53] Turro E., Greene D., Wijgaerts AThys C., Lentaigne C., Bariana T.K., Westbury S.K., Kelly A.M., Selleslag D., Stephens J.C., Papadia S., Simeoni I., Penkett C.J., Ashford S., Attwood A., Austin S., Bakchoul T., Collins P., Deevi S.V., Favier R., Kostadima M., Lambert M.P., Mathias M., Millar C.M., Peerlinck K., Perry D.J., Schulman S., Whitehorn D., Wittevrongel C., De Maeyer M., Rendon A., Gomez K., Erber W.N., Mumford A.D., Nurden P., Stirrups K., Bradley J.R., Raymond F.L., Laffan M.A., Van Geet C., Richardson S., Freson K., Ouwehand W.H., BRIDGE-BPD Consortium (2016). A dominant gain-of-function mutation in universal tyrosine kinase Src causes thrombocytopenia, myelofibrosis, bleeding, and bone pathologies. Sci. Transl. Med..

